# Antimicrobial profile of coagulase-negative staphylococcus isolates from categories of individuals at a neonatal intensive care unit of a tertiary hospital, Ghana

**DOI:** 10.11604/pamj.2023.44.92.37229

**Published:** 2023-02-16

**Authors:** Innocent Afeke, Kokou Hefoume Amegan-Aho, Joseph Adu-Amankwaah, Verner Ndudiri Orish, Graceful Lord Mensah, Hintermann Kobina Mbroh, Ibrahim Jamfaru, Abdul-Wahab Mawuko Hamid, Lennox Mac Ankrah, John Korbuvi, Anthony Ablordey

**Affiliations:** 1 Department of Medical Laboratory Sciences, School of Allied Health Sciences, University of Health and Allied Sciences, Ho, Ghana,; 2 Department of Pediatrics, School of Medicine, University of Health and Allied Sciences, Ho, Ghana,; 3 Department of Physiology, Xuzhou Medical University, Xuzhou, Jiangsu, China,; 4 Department of Microbiology and Immunology, School of Medicine, University of Health and Allied Sciences, Ho, Ghana,; 5 Department of Pediatrics, Ho Teaching Hospital, Ho, Ghana,; 6 Department of Obstetrics and Gynecology, Ho Teaching Hospital, Ho, Ghana,; 7 Department of Pharmacy, Ho Teaching Hospital, Ho, Ghana,; 8 Department of Bacteriology, Noguchi Memorial Institute for Medical Research, University of Ghana, Accra, Ghana

**Keywords:** Rational use, antibiotic, neonatal intensive care unit, epidemiology

## Abstract

**Introduction:**

we compared the antimicrobial resistance profile of young infants' clinical isolates (from blood samples) of Staphylococcus epidermidis and haemolyticus with those colonizing mothers, clinical staff, and students. Also, screened for resistance to the watch and reserve classified groups, antibiotics not prescribed in the Ho Teaching Hospital (HTH), Ghana.

**Methods:**

a cross-sectional study was conducted from March to June 2018 to determine the antimicrobial susceptibility of twenty-one antimicrobials for 123 isolates consisting of 54 S. epidermidis and 69 S. haemolyticus cultured from the participants. VITEK 2 was used for antimicrobial susceptibility testing. Staphylococcal species were identified using matrix-assisted laser desorption/ionization-time of flight (MALDI-TOF). Statistical analysis was done with Grad-Pad prism.

**Results:**

for S. epidermidis, clinical staff isolates have the highest methicillin-resistant (65%), followed by young infants' (50%) and mothers' and students' twenty-five percent each. Both young infants and clinical staff's Staphylococcus haemolyticus isolates have 100% methicillin-resistant, while mothers' and students' ones have 82% and 63%, respectively. We have identified resistance to one watch (teicoplanin), two reserves (tigecycline and fosfomycin) antimicrobial groups, and mupirocin, an unclassified group.

**Conclusion:**

identifying coagulase-negative staphylococci (CoNS) resistance to watch and reserve groups of antimicrobials in a non-previously exposed hospital calls for further studies to determine molecular mechanisms of resistance to these antimicrobials.

## Introduction

Microorganisms rapidly develop resistance to existing drugs; hence antimicrobial agents of new chemical groups are introduced, and the combined drugs are then used [1]. However, microorganisms' resistance grows faster than new antimicrobials are created. Considering the scale of the problem, the World Health Organization (WHO) has developed a document entitled “WHO Global Strategy for the Containment of Antibiotic Resistance” [2]. It states that excessive and inappropriate use of antibiotics is considered one of the major causes of the spread of resistance to antimicrobial drugs. Given this, in March 2017, the WHO Essential Medicines List (EML) Working Group classified antibiotics in the EML for children (EMLc) into three groups: access, watch, and reserve [3]. The access group generally contains narrow-spectrum antibiotics recommended as the first and second choice for most common clinical infection syndromes. They are used for commonly encountered susceptible pathogens and show lower resistance potential than antibiotics in the other groups. The watch group contains a generally broader spectrum of antibiotic classes. This group includes antimicrobials with higher resistance potential. It includes most of the highest priority agents among the critically important antimicrobials for human medicine [4] and antibiotics at a relatively high risk of selecting bacterial resistance. Antibiotics in the watch group are suggested to be prioritized as key targets of stewardship programs and monitoring. The reserve group consists of last-resort antibiotics for targeted use in multidrug-resistant infections. This group includes antimicrobial classes that should be reserved for treating confirmed or suspected infections due to multi-drug-resistant organisms. Antibiotics in the reserve group should be treated as “last resort” options; they should be accessible, but their use should be tailored to highly specific patients and settings when all alternatives have failed or are not suitable. To preserve their effectiveness, antibiotics in this group could be protected and prioritized as key targets of national and international stewardship programs involving monitoring and utilization reporting.

Antibiotics are the most frequently used medications in neonates. The neonatal intensive care unit (NICU) houses a naïve immunocompetent newborn who is highly susceptible to overwhelming infections. In the Ho Teaching Hospital´s (HTH´s) NICU and baby unit, early and decisive treatment with potent antibiotics for neonates with suspected infection is the preferred clinical doctrine owing to the fear of potentially disastrous consequences. The high associated mortality from infections leads neonatal care providers to initiate empirical antibiotic therapy in this hospital. However, antibiotics are often continued in clinical situations where a clear indication of benefit has not been demonstrated. As has already been reported, empiric antibiotic use for “rule-outs” contributes significantly to overall antibiotic use in neonatal units, making finding strategies for safe antibiotic restriction challenging, especially among preterm infants [5]. An ideal approach to early empirical antibiotic therapy would be one that accurately identifies and treats those at high risk while sparing those at low risk.

Coagulase-negative staphylococci (CoNS), primarily *S. epidermidis* and *S. haemolyticus*, are common etiologic agents in neonatal sepsis in low and middle-income countries (LMICs) [6]. There is epidemiological evidence that *S. aureus* strains resistant to some of the “Watch” and “Reserve” WHO classified antimicrobial groups are emerging from low- and middle-income countries (LMICs) where these antimicrobial agents are rarely used due to cost. Data on CoNS especially isolates in the NICUs, are mostly missing. Therefore, this study delivers insight into *S. epidermidis* and *S. haemolyticus* species' antimicrobial susceptibility patterns of some of the “Watch” and “Reserve” group antimicrobials and mupirocin for these bacterial species cultivated from a resource-limited hospital, where there were no prior exposures of these antimicrobials.

## Methods

**Study population and sample type collected:** our study was conducted at the Ho Teaching Hospital (HTH) of Ghana from March to June 2018. The study protocol was approved by the ethics committee of the University of Health and Allied Sciences (UHAS), Ho, Ghana. The study population includes 305 participants, which consist of 118 neonates (babies with the age of 0 - 28 days) and young infants (age range of 29 to 60 days), 68 mothers (transient residence), 59 clinical staff (residence), and 60 students (non-residence, control). The students were newly admitted to the university and had no contact with the hospital. Blood cultures were done for the babies, while nasal swab cultures were done for mothers, clinical staff, and students from the UHAS. We have previously described how these samples were cultured, stored, and shipped to Germany for further analyses [7].

**Bacterial isolates:** a total of 527 bacterial isolates were collected from the participants. Out of this, antimicrobial susceptibility testing was done for a selected 123 CoNS species consisting of 54 *S. epidermidis* and 69 *S. haemolyticus*.

**Identification of bacterial isolates:** all the bacterial isolates were identified in the Department of Infectious Diseases and Microbiology of the University of Lübeck, Germany, using the MALDI-TOF Biotyper® (Bruker Daltonik, Massachusetts, USA). The bacterial isolates were revived from glycerol-preserved stocks by seeding them on a 5% - 10% sheep blood agar plate and incubated at 37°C for 24 hours or until visible growth was observed on the plate. The bacterial isolates were spotted from a single colony onto a MALDI-TOF MS 48-well target plate per the manufacturer's instructions and identified by the machine. The results were confirmed with tuf gene sequence typing described by Hwang *et al*. [8]. Briefly, polymerase chain reaction (PCR) amplification of the tuf gene was performed on a C1000 Touch™ Thermal Cycler (BioRad) by applying a set of primers 5´-GCCAGTTGAGGACGTATTCT-3' and 5´-CCATTTCAGTACCTTCTGGTAA-3', which amplify a 412 bp fragment of the tuf gene. The PCR products were aliquoted (46μl) into 1 ml Eppendorf tubes, sealed, and shipped to GENEWIZ-Brooks Life Sciences, Leipzig, Germany, for tuf gene sequencing. Deoxyribonucleic acid (DNA) sequencing was done by the Sanger method. The obtained sequences of the tuf gene for each isolate were aligned separately by Molecular Evolutionary Genetics Analysis (MEGA 5) software and compared with all existing sequences of CoNS annotated in the GenBank database.

**Antimicrobial susceptibility testing:** overnight cultured bacterial isolates on blood agar media were observed for purity, and 0.5 McFarland concentrations were made with the manufacturer's diluent and checked with a benchtop turbidimeter. The inoculum was added to a VITEK 2 AST card according to the manufacturer's instructions. The reagent card was inserted into the machine for analysis. A purity check plate was performed by plating the diluted suspension on blood agar and incubating aerobically at 37°C overnight. The Minimum inhibitory concentrations (MICs) of 17 antibiotics were determined for these strains by VITEK 2® machine (bioMérieux, Durham, USA). S. aureus NCTC 12493 was included as a susceptible quality control strain. The MIC results were interpreted according to the 2018 European Committee on Antimicrobial Susceptibility Testing (EUCAST) clinical breakpoints. The MICs of mupirocin, teicoplanin, vancomycin, and tobramycin were also tested using gradient strips (E-test®; Liofilchem® s.r.l., Italy) using Mueller-Hinton E agar (bioMérieux SA, Strasbourg, France).

**Statistical analysis:** data were entered into an excel spreadsheet and imported into the Grad-Pad Prism 9.3.1.471 version. The frequencies and percentages were derived as part of descriptive statistics.

**Institutional review board statement:** the Research Ethics Committee of the University of Health and Allied Sciences (UHAS), Ghana, reviewed and approved this study with Protocol Identification Number UHAS-REC/A.2 [4] 17-18. Written approval was also obtained from the Ho Teaching Hospital (HTH) to use the facility for the study.

**Informed consent statement:** informed consent was obtained from all subjects involved in the study.

## Results

**Minimum inhibitory concentrations:** minimum inhibitory concentrations were obtained on an automated analyzer and then confirmed with Epsilometer. This study has identified resistance to one “Watch” antimicrobial group (teicoplanin), two “Reverse” groups (tigecycline and fosfomycin), and mupirocin, an unclassified group.

For *S. epidermidis* species, the highest methicillin resistance was identified among the isolates of the residence groups, clinical staff (65%), and young infants (50%), who represented the residents to the Ho Teaching Hospital. Rare resistance to the Fluoroquinolones antibiotic class was observed among the non-residence group (Table 1). Apart from fosfomycin, there was no resistance to the reserve group of antimicrobials tested. High resistance to tetracycline was identified for isolated colonizing mothers, who represent the transient residents of the HTH.

**Table 1:** percentage antimicrobial resistance for *Staphylococcus epidermidis* isolates

		Percentage resistance for participants’ types			
Classification	Antimicrobial agent	Babies' blood	Clinical staff's nasal mucosae	Mothers' nasal mucosae	Students' nasal mucosae
Methicillin-susceptibility	Cefoxitin	42	65	25	30
	Oxacillin	58	65	25	20
Access	Amikacin	8	25	17	5
	Ampicillin	30	67	50	20
	Clindamycin	25	35	33	10
	Gentamicin	50	35	17	5
	Penicillin G	100	83	90	83
	TRI-SUL	58	25	25	30
Watch	Erythromycin	33	40	25	5
	Ciprofloxacin	10	20	17	0
	Levofloxacin	20	43	18	0
	Moxifloxacin	20	36	17	0
	Teicoplanin	0	0	0	10
	Vancomycin	0	0	0	0
Reserve	Daptomycin	0	0	0	0
	Linezolid	0	0	0	0
	Fosfomycin	17	25	8	20
	Tigecycline	0	0	0	0
Unclassified	Mupirocin	8	0	0	0
	Tetracycline	67	40	91	40
	Rifampicin	17	35	18	20

TRI-SUL: trimethoprim/sulfamethoxazole

In the case of *S. haemolyticus* species, methicillin resistance was relatively high for all four groups with young neonates and clinical staff's isolates having 100% methicillin-resistant each. Teicoplanin resistance was seen among the isolates of young infants and mother groups but not in residence (clinical staff) and non-residence (students) groups. Isolates of the residence group have the highest resistance to the Fluoroquinolone class of antibiotics (Table 2). This staphylococcal species showed resistance to two reserve antimicrobial groups: fosfomycin and tigecycline. Both young infants and mothers' groups demonstrated the highest resistance to the antibiotic tetracycline.

**Table 2:** percentage antimicrobial resistance for *Staphylococcus haemolyticus* isolates

		Percentage resistance for participant types			
Classification	Antimicrobial agent	Babies' blood	Clinical staff's nasal mucosae	Mothers' nasal mucosae	Students' nasal mucosae
Methicillin-susceptibility	Cefoxitin	100	100	82	63
	Oxacillin	100	100	82	63
Access	Amikacin	10	18	13	5
	Ampicillin	90	63	80	50
	Clindamycin	36	50	36	13
	Gentamicin	73	63	55	25
	Penicillin G	91	90	90	80
	TRI-SUL	100	75	73	50
Watch	Erythromycin	55	75	46	25
	Ciprofloxacin	55	42	25	20
	Levofloxacin	36	63	36	13
	Moxifloxacin	36	63	36	13
	Teicoplanin	9	0	9	0
	Vancomycin	0	0	0	0
Reserve	Daptomycin	0	0	0	0
	Linezolid	0	0	0	0
	Fosfomycin	18	14	73	63
	Tigecycline	8	5	17	0
Unclassified	Mupirocin	8	5	0	0
	Tetracycline	82	63	82	38
	Rifampicin	27	50	18	5

TRI-SUL: trimethoprim/sulfamethoxazole

## Discussion

In our study, coagulase-negative staphylococci (CoNS) isolated from blood samples of young infants, nasal mucosae of mothers, clinical staff, and students were analyzed, and selected CoNS isolates were compared in terms of susceptibility and resistance. VITEK 2 was employed for antimicrobial susceptibility testing, and the resistance to some of the watch and reserve groups was confirmed with Epsilometer Test. CoNS were once thought incapable of inducing major clinical infections and were discarded as contamination when identified in cultures [9]. However, CoNS has been implicated in the development of neonatal sepsis in several studies, as babies treated in the NICU are regularly subjected to invasive and semi-invasive procedures that have a high risk of introducing bacteria into the bloodstream [10]. A high prevalence of healthcare workers and students have also been identified as nasal carriers of CoNS exhibiting different antimicrobial resistance profiles, including methicillin and multidrug-resistant [11,12]. CoNS have become one of the most common nosocomial infections resulting from patient's and procedure-related alterations, with *S. epidermidis* and *S. haemolyticus* being the most common species [6]. Our study revealed that *S. epidermidis* isolates from clinical staff have the highest methicillin resistance (65%), followed by young infants' (50%) and mothers and students' (25% each). These findings show that methicillin-resistant *S. epidermidis* is more prevalent in the HTH than in the normal community. A similar conclusion was obtained for *S. aureus*, with Bryna Warshawsky *et al*. identifying hospital contact as the single most important risk factor for methicillin-resistant Staphylococcus aureus (MRSA) acquisition [13]. *S. haemolyticus* isolates from newborn infants and clinical staff are 100% methicillin-resistant, while those from mothers and students are 82% and 63% methicillin-resistant, respectively. Similarly, a study carried out in a neonatal unit of a hospital in New Delhi reported a higher frequency of methicillin-resistant *S. haemolyticus* isolates than *S. epidermidis* isolated at the same time [14]. According to the authors, *S. haemolyticus* with three resistance patterns, including methicillin, were isolated from nasal cavities of mothers and staff in an adjacent maternity ward [14].

Microbes rapidly colonize neonates from the environment within the first week of life [15]. The use of central venous catheters (CVC), mechanical ventilation, parenteral feeding, and other invasive skin- or mucosa-breaching treatments significantly increases the risk of CoNS infection during this time [15,16]. CoNS are common occupants of the skin and mucous membranes; although a small percentage of neonates acquire CoNS through vertical transmission, the acquisition is usually horizontal. As a result, infants in the neonatal unit of hospitals get most of their microorganisms from the hospital environment, their parents, and the staff [15]. Antibiotic resistance in the skin and nasal-residing strains have been found to be low at birth but increase rapidly during the first week of hospitalization [17]. Previous studies have shown that transmission through the hands of staff and students at the hospital can result in endemic strains circulating for an extended period of time [18,19].

The rise of multidrug-resistant CoNS has made treating its associated diseases incredibly challenging. Studies have shown aside methicillin; CoNS are resistant to multiple antimicrobials, including tigecycline [20], teicoplanin [21], linezolid [20], and daptomycin [20]. In this study, we identified resistance of CoNS isolates to one watch (teicoplanin), two reserves (tigecycline and fosfomycin) antimicrobial groups, and mupirocin, an unclassified group. *S. epidermidis* isolates resistant to teicoplanin were seen among only students, while high resistance to tetracycline was identified for isolates colonizing mothers. Moreover, Adeapena *et al*. [22] reported tetracycline as the most widely administered antibiotic for animals in Ghana. In addition, over-the-counter antibiotics [23] and self-medication [24] are widespread among community members in Ghana. According to a Ghanaian study, community members insisted on purchasing specific antibiotics due to previous use and awareness of their effectiveness [23]. This could explain our present observation that tetracycline and teicoplanin resistance are more widespread in the Ho community than in hospitals. Regarding *S. haemolyticus*, teicoplanin resistance was seen among the isolates of young infants and mothers, while isolates from hospital staff had the highest resistance to the fluoroquinolone class of antibiotics. We have previously reported the antibiotics prescribed for young infants in the NICU of the HTH to include: ampicillin, amikacin, benzylpenicillin, cefotaxime, flucloxacillin, gentamicin and metronidazole [7]. Fluoroquinolones are not prescribed in the NICU hence we find it difficult to explain the high resistance of this class of antibiotics among the clinical staff of the HTH and calls for further research.

A study carried out by Mehri *et al*. showed a higher rate of multidrug-resistant isolates of *S. haemolyticus* and *S. epidermidis*, including resistance to teicoplanin. The authors concluded that teicoplanin might not be an effective first-line antibiotic for treating infections caused by these CoNS [21]. The current study discovered *S. haemolyticus* isolates that are resistant to tigecycline, a reserve group antibiotic defined by the WHO. Tigecycline belongs to the class of antibiotics known as glycylcyclines, which block protein synthesis and have action against a wide range of gram-positive and gram-negative organisms, including MRSA, vancomycin-resistant *Enterococcus spp*., and other difficult-to-treat pathogens [25]. As a derivative of minocycline, tigecycline prevents bacterial protein synthesis by attaching to the 30S subunit of the ribosome. However, it is not affected by classical mechanisms of resistance to tetracyclines, such as specific efflux pumps and ribosome protection [26,27]. Fosfomycin was another reserve group antimicrobial drug that showed resistance in *S. epidermidis* and *S. haemolyticus* isolates. Fosfomycin inhibits the formation of peptidoglycan precursors, causing bacterial cells to die. However, we will not go into detail about this antibiotic because we have not established its MICs with the Epsilometer Test, which is a limitation of our study.

## Conclusion

Taking together, this current study has drawn attention to the significant prevalence of methicillin-resistant CoNS in the NICU of the Ho Teaching Hospital, which may have been spread through nasal mucosae of mothers and clinical personnel. As a result, critical steps are required to eradicate these strains of CoNS in the NICU since these strains have been implicated in the development of neonatal sepsis. Also, the identification of CoNS resistance to watch and reserve groups of antimicrobials in a non-previously exposed hospital calls for further studies to determine molecular mechanisms of resistance to these antimicrobials.

### What is known about this topic

- CoNS, mostly S. epidermidis, and S. haemolyticusare the most frequent pathogens causing neonatal sepsis, especially late-onset sepsis in most neonatal intensive care units;
- Studies mostly conducted in developed countries have identified resistance of CoNS to some World Health Organization's classified reserve group of antimicrobials like tigecycline, daptomycin and linezolid;
- Resistance of CoNS isolates from developing countries, where reverse antimicrobials are hardly used, is a gap in research.

### What this study adds

- Resistance to tigecycline of S. haemolyticus isolates but not S. epidermidis was observed and called for further molecular studies to understand the mechanisms of resistance of S. haemolyticus isolate cultured in a non-previously exposed tigecycline hospital to tigecycline;
- This study observed high resistance to fosfomycin, a reverse-classified antibiotic, for S. epidermidis and S. haemolyticus isolates and calls for further research since fosfomycin is not a common antibiotic used in the study region;
- Higher methicillin resistance in S. haemolyticus is observed among individuals that had contact with a hospital facility than those who did not.
